# Supplementary material for: Causal Relationship between Aging and Anorexia Nervosa: A White-Matter-Microstructure-Mediated Mendelian Randomization Analysis
Source: Biomedicines. 2024 Aug 16;12(8):1874. doi: 10.3390/biomedicines12081874 (PMC11351342; doi:10.3390/biomedicines12081874)
Supplement: Supplementary file 1 [file biomedicines-12-01874-s001.zip › Supplementary Figure/Figure S4/Singe SNP plot of MR analysis for TL to ubm-b-1864.pdf]

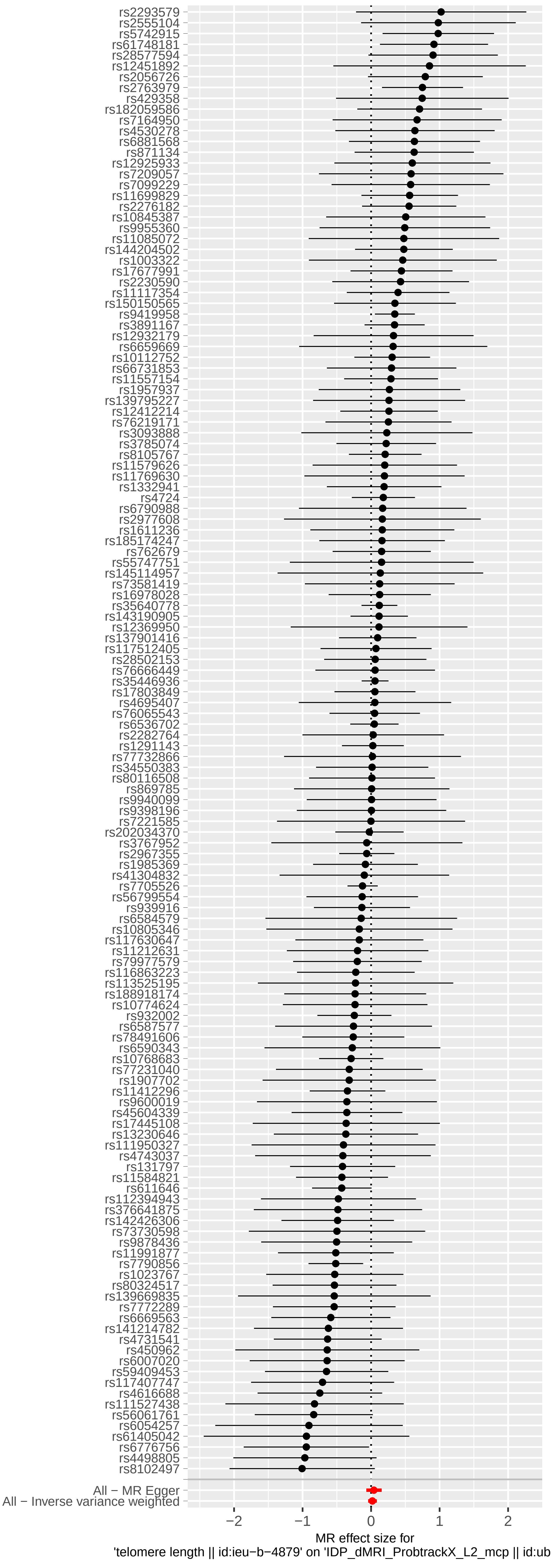
rs2293579
rs2555104
rs5742915
rs61748181
rs28577594
rs12451892
rs2056726
rs2763979
rs429358
rs182059586
rs7164950
rs4530278
rs6881568
rs871134
rs12925933
rs7209057
rs7099229
rs11699829
rs2276182
rs10845387
rs9955360
rs11085072
rs144204502
rs1003322
rs17677991
rs2230590
rs11117354
rs150150565
rs9419958
rs3891167
rs12932179
rs6659669
rs10112752
rs66731853
rs11557154
rs1957937
rs139795227
rs12412214
rs76219171
rs3093888
rs3785074
rs8105767
rs11579626
rs11769630
rs1332941
rs4724
rs6790988
rs2977608
rs1611236
rs185174247
rs762679
rs55747751
rs145114957
rs73581419
rs16978028
rs35640778
rs143190905
rs12369950
rs137901416
rs117512405
rs28502153
rs76666449
rs35446936
rs17803849
rs4695407
rs76065543
rs6536702
rs2282764
rs1291143
rs77732866
rs34550383
rs80116508
rs869785
rs9940099
rs9398196
rs7221585
rs202034370
rs3767952
rs2967355
rs1985369
rs41304832
rs7705526
rs56799554
rs939916
rs6584579
rs10805346
rs117630647
rs11212631
rs79977579
rs116863223
rs113525195
rs188918174
rs10774624
rs932002
rs6587577
rs78491606
rs6590343
rs10768683
rs77231040
rs1907702
rs11412296
rs9600019
rs45604339
rs17445108
rs13230646
rs111950327
rs4743037
rs131797
rs11584821
rs611646
rs112394943
rs376641875
rs142426306
rs73730598
rs9878436
rs11991877
rs7790856
rs1023767
rs80324517
rs139669835
rs7772289
rs6669563
rs141214782
rs4731541
rs450962
rs6007020
rs59409453
rs117407747
rs4616688
rs111527438
rs56061761
rs6054257
rs61405042
rs6776756
rs4498805
rs8102497
All − MR Egger
All − Inverse variance weighted
−2
−1
0
1
2
MR effect size for
'telomere length || id:ieu−b−4879' on 'IDP_dMRI_ProbtrackX_L2_mcp || id:ub
